# Epigenome-wide association study of smoking and DNA methylation in non-small cell lung neoplasms

**DOI:** 10.18632/oncotarget.11831

**Published:** 2016-09-02

**Authors:** Joshua R Freeman, Su Chu, Thomas Hsu, Yen-Tsung Huang

**Affiliations:** ^1^ Department of Epidemiology, Brown University, Providence, RI 02912, USA; ^2^ Department of Biostatistics and Epidemiology, School of Public Health and Health Sciences, University of Massachusetts, Amherst, Amherst, MA 01003, USA; ^3^ Department of Medicine, Brown University, Providence, RI 02912, USA; ^4^ Department of Biostatistics, Brown University, Providence, RI 02912, USA; ^5^ Institute of Statistical Science, Academia Sinica, Taipei 11529, Taiwan

**Keywords:** epigenetics, DNA methylation, non-small cell lung cancer, smoking

## Abstract

Tobacco smoke is a well-established lung cancer carcinogen. We hypothesize that epigenetic processes underlie carcinogenesis. The objective of this study is to examine the effects of smoke exposure on DNA methylation to search for novel susceptibility loci. We obtained epigenome-wide DNA methylation data from lung adenocarcinoma (LUAD) and lung squamous cell (LUSC) tissues in The Cancer Genome Atlas (TCGA). We performed a two-stage discovery (*n* = 326) and validation (*n* = 185) analysis to investigate the association of epigenetic DNA methylation level with cigarette smoking pack-years. We also externally validated our findings in an independent dataset. Linear model with least square estimator and spline regression were performed to examine the association between DNA methylation and smoking. We identified five CpG sites highly associated with pack-years of cigarette smoking. Smoking was negatively associated with methylation levels in cg25771041 (*WWTR1*, *p* = 3.6 × 10^−9^), cg16200496 (*NFIX*, *p* = 3.4 × 10^−12^), cg22515201 (*PLA2G6*, *p* = 1.0 × 10^−9^) and cg24823993 (*NHP2L1*, *p* = 5.1 × 10^−8^) and positively associated with the methylation level in cg11875268 (*SMUG1*, *p* = 4.3 × 10^−8^). The CpG-smoking association was stronger in LUSC than LUAD. Of the five loci, smoking explained the most variation in cg16200496 (R^2^ = 0.098 [both types] and 0.144 [LUSC]). We identified 5 novel CpG candidates that demonstrate differential methylation patterns associated with smoke exposure in lung neoplasms.

## INTRODUCTION

Tobacco is a major cause of many diseases, such as cardiovascular diseases [1, 2], pulmonary diseases [3, 4], cancers [5, 6] and most notably lung cancer [6, 7]. Based on 2012 estimates, worldwide 21% of individuals aged 15 or greater smoked tobacco products [8]. Globally, 1.42 million cancer deaths in 2000 were attributable to cigarette smoke exposure, 60% of which were due to lung cancer [9]. Lung cancer alone is also responsible for 12.4% of all new cancer cases and 17.6% of all cancer mortality [10]. In the U.S., lung cancer is the second most common cancer in both men and women, and the 5-year survival rate of lung cancer patients is 15.6%, which is much lower than other common types of cancers such as breast cancer (5-year survival rate 89.7%) [11] and prostate cancer (99.2%) [12]. Given the high incidence and poor prognoses for lung cancer, the high prevalence of smoking, and the lack of early diagnostic testing methods, it is critical that we understand the mechanisms by which tobacco smoking might cause lung cancer [10].

DNA methylation, the addition of a methyl group to DNA, may mechanistically regulate gene function [13, 14]. Differential DNA methylation, hypermethylation and hypomethylation of promoter-specific genes within CpG islands of tumor suppressor and proto-oncogenes, has been correlated with human cancers [15–21]. The triggers leading to aberrant epigenetic changes are poorly understood in the cancer genome, but those in blood have been implicated in cigarette smoking [22, 23]. Cigarette smoking has also been shown to be associated with genomic instability, which leads to DNA copy number alterations in the lung cancer genome [24]. For example, polycyclic aromatic hydrocarbons (PAHs), among other carcinogens in tobacco smoke have been well documented in altering DNA [25, 26]. However, less is known about how carcinogens may alter epigenetic machinery in the cancer genome.

The effects of cigarette smoke on DNA methylation range from modification of tissue methylation patterns to the development of disease [27–29]. A number of these studies have previously examined the link between smoking and DNA methylation [1, 18, 30–33], and while many candidate methylation loci have been identified, most studies used blood samples from patients. However, DNA methylation profiles are tissue specific, and blood tissue is unlikely to accurately represent lung cancer etiology [34–36]. It is important to examine site-specific DNA methylation to best understand how these disease-associated patterns may manifest *in vivo*. In this study, we used disease-appropriate neoplastic tissue from patients with lung squamous cell carcinoma (LUSC) and lung adenocarcinoma (LUAD) to study the effects of smoke exposure on DNA methylation. Because we use lung tumor tissue, which is directly exposed to cigarette smoke, rather than relying on proxy specimens such as blood, our study may provide greater insight into the link between smoking and methylation in lung cancer.

## RESULTS

In order to identify differentially methylated loci associated with smoking, we conducted an epigenome-wide analysis using a two-stage, discovery-validation approach in lung adenocarcinoma (LUAD) and lung squamous cell (LUSC) tissue samples of 511 subjects from The Cancer Genome Atlas (TCGA) database. Subjects were randomized into the discovery or validation analysis groups conditional on cell-type (LUAD or LUSC) in order to obtain a balanced distribution of each lung cancer tissue in the two subsets. Demographic characteristics for the 511 subjects by analytic group and smoking status (light or heavy smoking status, based on the median smoking pack-year (3.71)) are summarized in Table 1. None of the demographic and clinical characteristics were significantly different (Table 1) between the discovery (*n* = 326) and validation subsets (*n* = 185). Within-group distributions of sex, age and cell type of lung cancer were unbalanced between light and heavy smokers, i.e., heavy smokers were older, more likely to be male and to have squamous cell carcinoma (Table 1). Therefore, these potential confounders were adjusted either as covariates or stratifying factors in the epigenome-wide analyses.

**Table 1:** Demographic characters of sample by two-stage analysis and smoking status

	Discovery Set			Validation Set			Discovery vs. Validation		
Covariates	Light^†^ (*n* = 173)	Heavy^†^ (*n* = 153)	*P*-value	Light^†^ (*n* = 97)	Heavy^†^ (*n* = 88)	*P*-value	Discovery (*n* = 326)	Validation (*n* = 185)	*P*-Value
Age^*^	66.36 (58, 74)	70 (61, 73)	0.04	66 (59, 72)	67 (62, 72.25)	0.048	67 (60, 73)	66.36 (61, 72)	0.81
% of Male	91 (53%)	103 (67%)	0.007	54 (55.6%)	61 (69.3%)	0.055	194 (59.5%)	115 (62.2%)	0.56
Race^‡^									0.81
Black	10 (5.7%)	4 (2.6%)	0.37	6 (6.2%)	3 (3.4%)	0.68	14 (4.3%)	9 (4.9%)	
White	136 (78.6%)	124 (81%)		74 (76%)	69 (78.4%)		260 (79.8%)	143 (77.3%)	
Other	27 (15.6%)	25 (16.3%)		17 (17.5%)	16 (18.2%)		52 (15.95%)	33 (17.8%)	
KRAS mutation	5 (2.9%)	2 (1.3%)	0.55	4 (4.12%)	1 (1.13%)	0.43	7 (2.1%)	5 (2.7%)	0.92
EGFR mutation	5 (2.9%)	3 (2%)	0.86	1 (1.03%)	2 (2.27%)	0.94	8 (2.5%)	3 (1.6%)	0.76
PackYears^*^	3.367 (3.045, 3.58)	4.11 (3.93, 4.39)	2.20E-16	3.26 (3.05, 3.58)	4.08 (3.93, 4.39)	2.20E-16	3.71 (3.31, 4.11)	3.714 (3.26, 4.04)	0.71
% of ACA^‡^	105 (60.7%)	59 (38.6%)	1.00E-04	63 (65%)	41 (46.6%)	0.018	164 (50.3%)	104 (56%)	0.23
Smoking History^‡^	40 (23.1%)	61 (39.9%)	N/A	23 (23.7%)	36 (41%)	0.02	101 (31%)	59 (32.2%)	0.16

* Median (1st, 3rd quartiles).

† Light and Heavy were determined by a median cutoff for the smoking packyears.

‡ *P*-values were calculated using Chi-squre tests. All other *p*-values were calculated using Student's *t*-test.

### EWAS identification of differentially methylated sites associated with smoking

**Figure 1:**
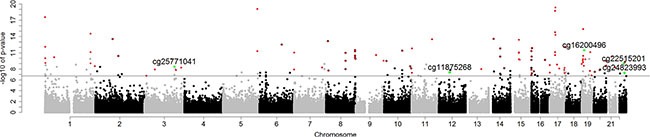
Manhattan plot of *p*-values for internally validated (No. of loci = 98) and externally validated (No. of loci = 5) CpG sites by chromosome Bonferroni genome-wide significance (6.73) is represented as a horizontal solid line. Red dots are internally validated sites; green dots are internally and externally validated sites.

In the first stage, an epigenome-wide association scan was conducted in the discovery subset to test the relationship between smoking and DNA methylation at each CpG site, with adjustments for cell-type, *EGFR* and *KRAS* mutation status, age, sex, and race. We identified 263 out of 271,316 CpG sites, which were significant at FDR<0.05 in this stage. Of these, we identified 98 CpG loci which had 1) consistent directions of effect in both analytic stages, 2) *p*-value < 0.001 in the validation stage (Supplementary File S1) and were thus considered internally validated (Figure 1).

### Externally validated candidate CpG loci

Further analysis of the 98 internally validated CpG sites in an external dataset, GSE56044 from the Gene Expression Omnibus database (demographic characteristics are summarized in Supplementary Table S1), identified five CpG sites with independently replicated signals for association between smoking and DNA methylation. As shown in Table 2, the five externally validated sites and their associated genes were: cg25771041 in WW domain containing transcription regulator 1 gene (*WWTR1*; pooled *p*-value = 3.63 × 10^−9^, external *p*-value = 0.046), cg11875268 in single-strand-selective monofunctional uracil DNA glycosylase gene (*SMUG1*; pooled *p*-value = 4.28 × 10^−8^, external *p*-value = 0.0017), cg16200496 in nuclear factor 1 x-type gene (*NFIX*; pooled *p*-value = 3.4 × 10^−12^, external *p*-value = 0.0344), cg22515201 in phospholipase A2 group VI gene (*PLA2G6*; pooled *p*-value = 1.04 × 10^−9^, external *p*-value (in LUAD) = 0.016), cg24823993 in NHP2-like protein 1 gene (*NHP2L1*; pooled *p*-value = 5.13 × 10^−8^, external *p*-value (in LUAD) = 0.047). We noted that the three most significant CpG sites that were validated internally: cg16579555 (pooled *p* = 4.2 × 10^−20^; located within *RNF135* [ring finger protein 135]), cg00032419 (pooled *p* = 1.8 × 10^−19^; located within *TP53I13* [tumor protein P53 inducible protein 13]) and cg16654732 (pooled *p* = 8.1 × 10^−20^; located within *FGF18* [fibroblast growth factor 18]) seemed biologically interesting but were not validated by the external data.

**Table 2:** Results of the 5 externally validated CpG sites

CpG Descriptors				Discovery			Validation		Pooled		Without documented KRAS or EGFR Mutations		With Adjustment for Cancer Cell Stage		External Validation			
CpG ID	Ch	Symbol	Location	Beta*	*P*-value	FDR	Beta*	*P*-value	Beta*	*P*-value	Beta*	*P*-value	Beta*	*P*-value	Beta^†^(binary)	*P*-value(binary)	Beta^‡^(ordinal)	*P*-value(ordinal)
cg25771041	3	WWTR1	149376042	–0.353	1.48E–05	0.023	–0.463	8.28E–05	–0.387	3.63E–09	–0.393	6.71E– 09	–0.383	5.42E–09	–1.13	0.046	–0.292	0.357
cgl1875268	12	SMUG1	54576025	0.849	2.92E–05	0.035	0.978	3.31E– 04	0.875	4.28E–08	0.866	1.12E–07	0.876	4.54E–08	2.23	0.0017	0.67	0.097
cgl6200496	19	NFIX	13107141	–0.634	6.71 E–07	0.003	–0.878	2.27E–06	–0.721	3.40E–12	–0.742	4.40E–12	–0.721	3.94E–12	–2.23	0.0344	–0.324	0.585
cg22515201	22	PLA2G6	38577827	–0.929	1.64E–06	0.006	–0.649	9.82E–04	–0.859	1.04E–09	–0.851	2.09E–09	–0.851	6.64E–10	–2.33	0.0947	–1.01	0.193
cg24823993	22	NHP2L1	42085003	–0.406	3.42E–05	0.038	–0.244	6.52E–06	–0.351	5.13E–08	–0.354	1.02E–07	–0.356	3.58E–08	–3.06	0.0513	–1.29	0.143

cg25771041 is in CpG island; cgl 1875268 is not in CpG island; cgl 6200496, cg22515201 and cg24823993 are all in CpG island and promoter associated.

* Beta here is the difference in methylation M-value per one-unit increase in log-transformed smoking pack-years.

† Beta here is the difference in methylation M-value comparing ever smokers with never smokers.

‡ Beta here is the difference in methylation M-value between current smokers and former smokers as well as between former smokers and never smokers.

Higher smoking exposure was associated with decreased methylation at cg25771041, cg16200496, cg22515201, and cg24823993, and increased methylation at cg11875268. The direction of association for these loci was consistent across the internal discovery and validation subsets, as well as in the external validation analyses. Adjusting for cancer stage level (I-IV) also resulted in stronger statistical significance for all externally validated CpG loci (Table 2). Associations between CpG site methylation and RNA expression of their associated genes were also assessed for subjects who also had expression data available; only cg25771041 at *NHP2L1* demonstrated a significant association between methylation and RNA expression (Supplementary Table S4).

### DNA methylation signal profiles across candidate genes

**Figure 2:**
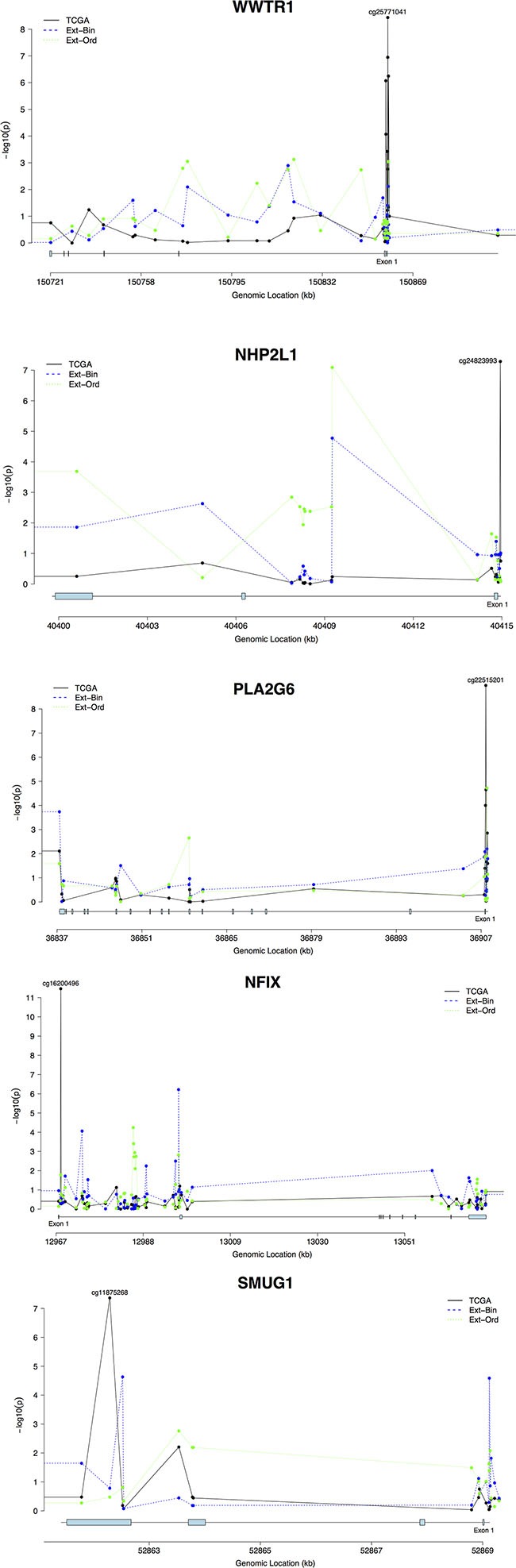
Methylation signal profiles for genes with externally validated methylation sites **(A. *WWTR1*; B. *NHP2L1*; C. *PLA2G6*; D. *NFIX*; E. *SMUG1*)**. The analyses are plotted by genomic datasets. TCGA represents the TCGA dataset, and External-Binary (Ext-Bin) and External-Ordinal (Ext-Ord) represent the external validation analyses conducted in the GSE56044 dataset using binary and ordinal categorizations of smoking status. Signal strength is plotted via transformed *p*-values (-log10(p)) by genomic location (Mb) for each gene.

DNA methylation profiles for all genes containing externally-validated CpG sites were mapped to explore whether any interesting methylation patterns could be discerned across the genes (Figure 2). In *WWTR1*, *NHP2L1*, and *PLA2G6*, evidence of significant methylation near the transcription start sites (TSS) was present based on the pooled TCGA analyses, with multiple significant CpG hits in the TSS neighborhoods in *WWTR1* and *PLA2G6*. In *NFIX*, no significant methylation signals other than the externally validated finding at cg16200496 were present. Finally, in *SMUG1*, several significant methylation loci were present across the gene body but not in the TSS.

Several CpG loci in *WWTR1* with significant association with smoking localize to a CpG island either within 200–1500 base pairs of the transcription start site (TSS), or in the 5′ untranslated region (UTR), depending on the isoform. The most significant association signal between DNA methylation and cigarette smoking in *WWTR1* occurred at cg25771041 (Figure 2A). cg24823993 with the most significant signal between smoking and DNA methylation in *NHP2L1*, locates within 200 bp of the TSS (Figure 2B). The binary-smoking external validation analyses also identified cg24823993 as a marginally significant methylation site. cg22515201 in a CpG island within 200 bp of the TSS in *PLA2G6* is the most significant CpG locus in the gene associated with cigarette smoking. The analyses also identified several highly significant methylation loci at the TSS and near exon 1 of *PLA2G6* (Figure 2C). The analyses of CpG loci within *NFIX* identified highly significant association with smoking in exon 1 with the most significant signal in cg16200496, but no other regions demonstrate strong enrichment (Figure 2D). The association with smoking was more prominent in the fourth exon (cg11875268) of *SMUG1* (Figure 2E).

### Dose-response relationships between smoking and CpG methylation

**Figure 3:**
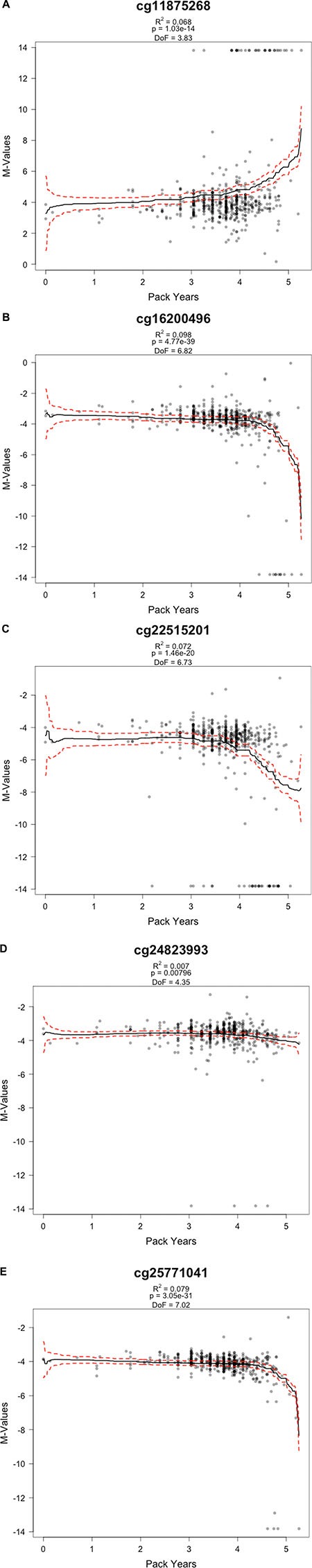
Dose-response relationships for externally validated CpG sites by M-values (logit transformed beta values) and smoking in pack years The plots are based on generalized additive models with penalized spline using thin plate smoothing basis. Degree of Freedom (DoF) is provided for each plot. The solid black line represents the linear spline model of the change in M-value by pack years (smoking). The red, dotted line represents the upper and lower 95% confidence bounds. (**A**) cg11875268 in *SMUG1*; (**B**) cg16200496 in *NFIX*; (**C**) cg22515201 in *PLA2G6*; (**D**) cg24823993 in *NHP2L1*; (**E**) cg25771041 in *WWTR1*.

To examine potential dose-response relationships between pack-years of cigarette smoking and methylation status, linear models with penalized spline (thin-plate regression spline) were constructed for the five validated CpG sites (Figure 3). There were statistically significant negative associations for all CpG sites as pack-years increased (cg25771041 [in *WWTR1*; *p* = 3.1 × 10^−31^, R^2^ = 0.079], cg16200496 [in *NFIX*; *p* = 4.8 × 10^−39^, R^2^ = 0.098], cg22515201 [in *PLA2G6*; *p* = 1.5 × 10^−20^, R^2^ = 0.072], cg24823993 [in *NHP2L1*; *p* = 0.008, R^2^ = 0.007]) except in cg11875268 (in *SMUG1*; *p* = 1 × 10^−14^, R^2^ = 0.068) (Figure 3A). Variation in cg22515201 (*PLA2G6*) was explained the most by smoking pack-years with a marginal R^2^ = 0.098. Marginal R^2^ values between smoking and methylation at the five loci were quite low (ranging from 0.007 to 0.098), suggesting that cigarette smoking alone does not fully explain changes in methylation status at these loci, and further implying the likely presence of other environmental and/or genetic determinants for epigenetic variations.

To check the robustness of the dose-response analyses, we also conducted sensitivity analyses which 1) removed extreme *M*-values and 2) substituted extreme *M*-values with less extreme values (the detectable largest/smallest values; Supplementary Figure S2). All validated CpG sites retained significant dose-response relationships with smoking in the analyses where extreme outliers were removed except cg11875268 (*p* = 0.052). All validated CpG sites retained significant dose-response relationships with smoking in the substitution analyses.

### Cell-type restricted sub-analyses and sensitivity analyses

To identify cancer-specific methylation markers, sub-analyses were conducted by stratifying by cell-type and obtaining cell-type-specific estimates for the 98 internally validated CpG loci in the internal discovery, validation, and pooled samples respectively (Supplementary Files S2 and S3). The cell-type restricted subanalyses were then conducted in GSE56044 in an equivalent manner. In the internal subgroup analyses, the cell-type specific estimates retained directions of effect consistent with the effect estimates from the main analyses for all five externally validated loci. Interestingly, the strength of association was consistently more significant in the LUSC subgroup, with the strongest signal originating from cg16200946 in *NFIX* (β = −1.20, *p* = 6.6 × 10^−10^, R^2^ = 0.144) (Supplementary File S3 and Supplementary Figure S1D). The cell-type specific association between DNA methylation of the five externally validated loci (cg25771041, cg11875268, cg16200496, cg22515201 and cg24823993) and smoking was presented in Supplementary Tables S2 and S3, and their dose-response relationship was presented in Supplementary Figure S1. Further discussion of the cell-type-specific EWAS is available in the supplement.

Sensitivity analyses were conducted by reanalyzing the main, cell-specific, and external validation datasets after removing cases with KRAS or EGFR mutation from our analytical model. Each sensitivity analysis yielded consistently similar results to those from the main, cell-specific, and external validation analyses, respectively (Table 2), providing compelling support for the biological plausibility of our reported findings.

## DISCUSSION

Our results identify five candidate methylation loci which may be influential in how smoking modifies DNA methylation, and thus also the development of lung cancer. Of our 98 internally validated CpG sites, five were externally validated in an independent data set: cg16200496 (*NFIX*), cg25771041 (*WWTR1*), cg11875268 (*SMUG1*), cg22515201 (*PLA2G6*), and cg24823993 (*NHP2L1*). Four of these loci localized near transcription start sites or within the first exons of their genes (*WWTR1*, *NFIX*, *PLA2G6* and *NHP2L1*), and all four demonstrated negative associations between smoking and methylation status. We examined cancer stage as a confounder for smoking and DNA methylation. We utilized a missing indicator method to adjust for the available cancer stage information while keeping all subjects in the analyses. The analyses showed that our externally validated loci were more statistically significant than without adjustment. This suggests that smoking may alter neoplasm development with increased pack years being associated with higher cancer stage.

Among the five genes, a number have been previously implicated in smoking and lung cancer disease pathways. *WWTR1* (also known as *TAZ*) is a well-described oncogenic transcriptional co-activator in many cancers including breast, liver, colon, thyroid, and lung [37, 38]. It is a part of the Hippo signaling pathway which is highly conserved in mammals and is thought to disrupt cell contact inhibition, an attribute commonly lost in cancer cells [39]. In one study, tumor-propagating cells were found to have gene expression signatures enriched for genes in the Hippo signaling pathway. Further experimentation with *WWTR1*/*TAZ* knockdowns resulted in decreased lung tumor progression, while constitutively active *WWTR1*/*TAZ* was found to be sufficient to drive lung tumor progression [40]. Additionally, higher *TAZ* expression levels in lung tumors have been shown to be predictive of worse prognoses [41, 42].

Another significant cancer-associated gene in our analyses was *SMUG1*, a glycosylase that removes damaged uracil in the base excision repair pathway [43]. The base excision repair pathway plays a critical role in removing oxidized and methylated bases from DNA, and has been implicated in a number of cancer subtypes including gastric, renal, lung, and colorectal cancers [44]. Importantly, *SMUG1* has also been hypothesized to play a critical role in nucleic acid repair in lung fibroblasts suffering from cigarette-smoke induced oxidative stress [45]. This offers compelling biological implications for our finding of significant association between smoking and methylation status at cg11875268.

A third well-established cancer gene in our significant results was *NFIX* (Nuclear Factor I/X (CCAAT-binding transcription factor)). *NFIX* is a member of a family of transcription factors that are involved in regulating the transcriptional activity of genes [46, 47]; and has been involved in cancer progression in a number of cancers including breast and esophageal [46, 48]. In breast cancer, *NFIX* may interact with methyl-CpG binding protein 2 (MeCP2), an important epigenetic regulator, to suppress Z-DNA-mediated transcriptional suppression, thus enabling the overexpression of *ADAM-12*, a prominently up-regulated, metastasis-promoting protein in many cancer types.[49] In esophageal cancers, the down-regulation of *NFIX* allows for microRNA miR-1290 to promote tumor proliferation, migration and metastasis [46].

The roles of *PLA2G6* and *NHP2L1* are not well understood in LUSC and LUAD. One candidate gene pathway analysis identified *PLA2G6*, a member of the cell cycle pathway, as bearing a statistically significant single nucleotide polymorphism associated with lung cancer risk [50]. *NHP2L1* is less understood, but is important in cell viability in yeast models and as an RNA-binding protein, specifically a small nucleolar RNA-protein complex, in eukaryotic models [51, 52]. Our study is the first to link these CpG sites to LUAD and LUSC in humans. Further research should examine the roles of these CpG sites in carcinogenesis given the strength of this finding.

In the internally validated-only CpG loci, cg16654732 was our strongest signal (pooled *p*-value = 8.1 × 10^−20^). This site localizes within 200bp of the TSS of gene *FGF18*, which was found to be down regulated in Italian LUAD cases compared to normal lung tissue [53]. In the present analyses, we found that methylation at cg16654732 was negatively associated with smoking, where higher pack years corresponded with lower methylation levels. In addition, cg13204512 and cg16579555 (within *RNF135*) were strongly, negatively associated with smoking pack-years (pooled *p*-value = 4.8 × 10^−15^ and 4.2 × 10^−20^, respectively). *RNF135* gene has been well studied in malignant peripheral nerve sheath tumors and lymphoblastic leukemia [54, 55], but our study is the first to link these loci to smoking in non-small cell lung tumors. The *TP53I13*, TP53 inducible gene 13 also had two strong signals from the analyses of TCGA data (cg00032419, *p* = 1.8 × 10^−19^ and cg00265578, *p* = 1.6 × 10^−15^). TP53-inducible genes have been well documented to control many biological processes including cell cycle control, apoptosis, and DNA repair and may function to inhibit cancer progression [56]. The internal analysis showed these two CpG loci were negatively associated with smoking dosage, which may indicate these genes were active in the neoplastic tissue. Despite the strength of the association in our analyses, cg16654732, cg13204512, cg16579555, cg00032419 and cg00265578 were not found to be statistically significant in the external dataset suggesting this finding may have mechanistic heterogeneity and may not be generalizable to other studies.

For the five externally validated CpG sites, the binary effect estimates were larger than the ordinal effect estimates. It is difficult to distinguish whether there is a dose-response increase from never- to ever- and then current-smokers or a plateau effect that ever-/current-smokers share similar effects. While years since quitting smoking may help address the issue, such information was not collected in TCGA data. Research by van Osch et al. (2016) suggests that a plateau effect of smoking on bladder cancer risk and that heavy smokers are at high risk regardless of the timing of cessation for given packyears [57].

In considering the importance of tissue sample location, we utilized LUSC and LUAD neoplasms. Other studies have used whole blood samples in their EWAS, but few have used neoplasm site-specific analyses. In one site-specific analysis, Teschendorff et al. used buccal cells in their EWAS of epithelial cancers. However, we were unable to replicate their findings [58]. This lends credibility to the notion that the effect of smoking on differential methylation is site-specific. Despite smoking carcinogen presence in buccal cells, it seems there may be a different mechanism through which smoke may act on methylation profiles in different genes in different environments.

There were many strengths in our study analysis. We had a large sample size (*n* = 511), improving the power of our EWAS interrogation of 271,316 CpG sites. Furthermore, we obtained data collected from an appropriate target tissue—lung neoplasms. Many studies have performed EWAS using blood samples, but blood samples are not the ideal tissue to measure carcinogenesis in lung tissue and can only serve as a proxy for lung cancer rather than a direct sample of the cancer itself. Because our study used lung neoplasms, we were able to directly assess the methylation patterns of CpG sites within LUSC and LUAD. Cell-type specific analyses were also conducted to better understand differential methylation due to smoking in adenocarcinoma and squamous cell carcinoma separately. Not only were our results internally validated within the TCGA data, we were also able to replicate some of our findings in the external GSE56044 GEO dataset. The multi-step internal and external validation conducted in our analysis lowers the likelihood of obtaining false-positive CpG hits.

Although our study has strengths, there are some limitations. While our data suggest that smoking regulates methylation patterns in neoplasms, we are unable to directly link smoking and lung cancer. Our sample is based solely on patients with LUSC or LUAD neoplasms; we lack healthy participants and are therefore could not assess methylation patterns in the lung tissue of healthy smokers. Therefore, we were unable to directly assess lung cancer etiology. Furthermore, it is unclear whether the validated CpG sites had significant methylation profiles solely due to smoking or because of other unmeasured factors. Dichotomous cell-type adjustment of adenocarcinoma and squamous cell carcinoma may be coarse, but it is standard and widely available information in clinical practice. Without depending on more costly pathological profiling, the identified methylation biomarkers may have better translation utility. Alternatively, one may adjust for inferred cell mixture based on a bioinformatics algorithm [59] if the research interest is in the epigenetic association with smoking within a homogeneous cell population. Here we are more interested in such an association within a patient with non-small cell lung cancer, lung adenocarcinoma or lung squamous cell carcinoma.

We note that the five CpG hits in the external validation analyses were significant after adjustment of multiple comparisons. However, since 1) these sites were internally validated and 2) the external validation analyses did not stand alone, we were less concerned about the potential false positive due to multiple comparisons. Furthermore, the external validation data measured categorical smoking status rather than smoking pack-years, which may render the external validation less power and thus being non-ideal for conservative multiplicity adjustment.

Our data indicate that CpG sites in *WWTR1*, *NFIX*, *PLA2G6*, *NHP2L1* and *SMUG1* have differential methylation in LUAD and LUSC neoplasms. These internally and externally validated CpG sites may give insight into the mechanism by which smoking may cause lung cancer. Additional research should focus on how these CpG sites are mechanically altered after repeated smoke exposure and if there are hierarchical interactions with microRNA and proteins from other CpG sites.

## MATERIALS AND METHODS

### Study sample

Subject data (*n* = 820) were obtained from The Cancer Genome Atlas (TCGA) (https:// tcga-data.nci.nih.gov/tcga), a collaborative project between the National Cancer Institute (NCI) and the National Human Genome Research Institute (NHGRI) that curates publicly available cancer datasets which have been comprehensively genotyped and assayed. Specific information on sample quality control has been previously reported [60]. We selected sample based on the availability of 1) epigenome-wide DNA methylation data from untreated neoplastic LUAD and LUSC tumor cells (classified as stages I–IV), and 2) clinical measures of smoke exposure. Subjects with missing methylation data (*n* = 139) and missing smoking measures (*n* = 170) were excluded, resulting in an analytical sample of 511 (268 LUAD and 243 LUSC) subjects.

### Data processing

Key clinical and demographic variables of interest were re-categorized for analysis: smoking exposure in pack years, sex, age, race, *KRAS* mutation, *EGFR* mutation, and cell-type. Pack-years, defined as the packs of cigarette smoked multiplied by the duration of smoking, was log transformed due to skewness. Both *KRAS* and *EGFR* mutation types were re-categorized into binary variables based on whether any mutations were present: the presence of any mutation (e.g., exon 19 deletion, L858R, and others) or not. Race was re-categorized as a nominal, categorical variable with race designations “white”, “black”, and “other.” Missing information in age was imputed with the median values of age in the full sample.

Level 3 methylation data assayed on the Illumina Infinium Human Methylation 450K in LUSC and LUAD neoplasms were obtained from TCGA database. All CpG sites located in sex chromosomes were discarded, retaining only autosomal sites. We adjusted for batch effects using the ComBat method in the Surrogate Variable Analysis (sva) package from Bioconductor CpG sites with low variance were filtered out based on the first quartile of the variance for all autosomal CpG sites (σ = 0.147) [61]. After quality-control, 271,316 CpG sites were retained for EWAS analysis.

### Statistical analysis

#### Internal discovery and validation analysis

Candidate methylation loci were identified and validated using a two-stage approach by randomizing all subjects (*n* = 511) into discovery (*n* = 326) and validation (*n* = 185) subsets. Randomized assignment was performed conditional on cell-type (LUAD or LUSC) in order to obtain a balanced distribution of each lung cancer tissue in the two subsets. Potential confounders were stratified or treated as covariates in regression analyses. In the first stage, an epigenome-wide association scan was conducted in the discovery subset using a linear model to test the relationship between DNA methylation and smoking at each CpG locus, with adjustments for cell-type, *EGFR* mutation status, *KRAS* mutation status, age, sex, and race. To adjust for multiple comparisons, we then applied a false discovery rate (FDR) < 0.05 threshold in the discovery analyses using the FDRtool R package [62]. CpG sites surviving the FDR < 0.05 threshold in the discovery stage were then re-analyzed in the validation subset using the same model specifications as in the discovery stage, and those loci with a validation *p*-value < 0.001 were retained for further cell-type-specific sub-analyses. All candidate loci surviving both the discovery and validation analyses were considered internally validated. Internally validated sites were then reassessed in the full study sample (i.e. the combined discovery and validation subsets) to obtain the final pooled *p*-values. Further adjustment for cancer stage and cancer stage missingness was conducted for the externally validated CpG sites using the missing indicator method [63].

#### External validation analysis

To validate our findings in an independent dataset, candidate CpG sites identified in the two-stage analyses were re-analyzed in the GSE56044 dataset (*n* = 124), which was obtained from the NCBI's Gene Expression Omnibus (GEO) database (http://www.ncbi.nlm.nih.gov/geo/query/acc.cgi?acc=GSE56044). All covariates were operationalized as in the main analysis with one exception: in the GSE56044 dataset, smoking status was recorded as a categorical variable (never smoker, current smoker, and former smoker) rather than continuously in pack-years. To assess any underlying dose-response relationships, we recoded smoking as an ordinal variable (never smoker = 0, former smoker = 1, current smoker = 2) and as a binary variable (non-smoker = 0, ever-smoker = 1) in GSE56044. The model specification used for validation in the external dataset was identical to the model used in our main analyses. Finally, we also conducted sensitivity analyses to check the robustness of our dose-respond trends by re-analyzing the relationships after 1) removing extreme M-values and 2) substituting extreme M-values with less extreme values (the detectable largest/smallest values; Supplementary Figure S2).

#### Cell-Type specific sub-analyses and sensitivity analyses

To assess association between smoking and DNA methylation that may be specific to cell-type, we conducted sub-analyses restricting to cell-type. Cell-type-specific estimates for the 98 internally validated CpG loci were obtained in the pooled samples (Supplementary Files S2 and S3). The cell-type restricted subanalyses were then also conducted in GSE56044 in an equivalent manner. To examine the robustness of our findings, further sensitivity analyses were conducted by comparing estimates obtained by including vs excluding subjects with documented *EGFR* (*n* = 11) or *KRAS* (*n* = 12) mutations within the main and cell-type-specific analyses.

## SUPPLEMENTARY MATERIALS








