# Supplementary material for: Epigenome-wide association study of smoking and DNA methylation in non-small cell lung neoplasms
Source: Oncotarget. 2016 Sep 2;7(43):69579–91. doi: 10.18632/oncotarget.11831 (PMC5342499; doi:10.18632/oncotarget.11831)
Supplement: Supplementary file 1 [file oncotarget-07-69579-s001.pdf]

# Epigenome-wide association study of smoking and DNA methylation in non-small cell lung neoplasms

## Supplementary Materials

### Cell-type specific sub-analysis

To assess possible cancer cell type-specific methylation, we conducted a cell type specific sub-analysis examining of the 98 internally and the 5 externally validated sites from the main analysis in a squamous cell carcinoma-only ($n$ = 243) and adenocarcinoma-only ($n$ = 268) model.

Overall, the results were consistent between the combined and cell type specific analyses. The five externally validated sites in the adenocarcinoma-specific analysis had pooled effect estimates in the same direction as the main analysis (Supplementary Table S2). The strongest signal was cg22515201 (*PLA2G6*; beta = −0.87, pooled $p$-value = $2.23 \times 10^{-6}$, external $p$-value = 0.016). The squamous cell carcinoma-only sub-analysis also showed similar results compared to the main analysis with changes in the effect magnitude. From the internally validation analysis, the strongest statistically significant CpG site was cg16200496 (*NFIX*; beta = −1.20, pooled $p$-value = $6.57 \times 10^{-10}$, external $p$-value = 0.27) (Supplementary Table S3). We noted that the external validation for squamous cell carcinoma-only analyses was not stable due to the limited sample size. Furthermore, there were no cases with *EGFR* or *KRAS* mutations in the squamous cell restricted analysis.

We investigated dose-response relationship for the five externally validated CpG sites (Supplementary Figure S1). Descriptions of trend and statistical significance were provided in Supplementary Figure S1. Briefly, the directionality of effect estimate is identical between dose-response relationships in LUAD and LUSC. However, the effect estimate was statistically significantly stronger in LUSC except for cg22515201 (Supplementary Figure S1E and S1F) compared to the same sites in LUAD suggesting differential mechanistic processes in this cell type. Cigarette smoking explained the most variation of cg16200496 in LUSC ($R^2$ = 0.144). Similarly to the main analysis, $R^2$ values of other CpG sites were consistently low, suggesting that epigenetic variation is not adequately explained by smoking alone.

## Supplementary Table S1: GSE56044 demographics by smoking status

| Covariates | Never (*N* = 20) | Former (*N* = 54) | Current (*N* = 32) | All (*N* = 106) |
|---|---|---|---|---|
| Age* | 78.00 (73, 80) | 70 (64, 74) | 62 (56, 69) | 69.50 (62, 75) |
| Gender** | 6 (30.0%) | 31 (57.4%) | 12 (37.5%) | 49 (46.2%) |
| KRAS | 0 (0%) | 13 (24.1%) | 11 (34.4%) | 24 (22.6%) |
| EGFR | 8 (40%) | 4 (7.4%) | 0 (0%) | 12 (11.3%) |
| Celltype* | 19 (95% A) | 39 (72.2% A) | 25 (78.1% A) | 83 (78.3% A) |

* Median, (1st, 3rd Quartiles).
**Sex Descriptors refer to Males.
***Cell Type refers to Adenocarcinoma.
Race data not available.

## Supplementary Table S2: Adenocarcinoma-specific analyses for the five externally validated CpG loi

| | Pooled | | No Mutations | | External Validation | | | |
|---|---|---|---|---|---|---|---|---|
| CpG Site | Beta | *P*-value | Beta | *P*-value | Beta (binary) | *P*-value (binary) | Beta (ordinal) | *P*-value (ordinal) |
| cg25771041 | −0.236 | 1.52E-03 | −0.237 | 0.003 | −1.238 | 0.0632 | −0.358 | 0.3697 |
| cg11875268 | 0.473 | 1.20E-05 | 0.435 | 2.51E-05 | 1.545 | 0.0428 | 0.455 | 0.3197 |
| cg16200496 | −0.443 | 1.04E-04 | −0.456 | 1.59E-04 | −2.302 | 0.0286 | −0.840 | 0.1824 |
| cg22515201 | −0.872 | 2.23E-06 | −0.864 | 3.82E-06 | −1.842 | 0.1082 | −1.622 | 0.0160 |
| cg24823993 | −0.349 | 6.03E-06 | −0.357 | 1.23E-05 | −3.112 | 0.0430 | −1.813 | 0.0471 |

## Supplementary Table S3: Squamous cell carcinoma-specific analyses for the five externally validated CpG loi

| | Pooled | | No Mutations | | External Validation | | | |
|---|---|---|---|---|---|---|---|---|
| CpG Site | Beta | *P*-value | Beta | *P*-value | Beta (binary) | *P*-value (binary) | Beta (ordinal) | *P*-value (ordinal) |
| cg25771041 | –0.682 | 3.17E-08 | – | – | NA | NA | NA | NA |
| cg11875268 | 1.656 | 5.00E-06 | – | – | 10.969 | 1.50E–270 | 1.628 | 0.0675 |
| cg16200496 | –1.204 | 6.57E-10 | – | – | –0.860 | 0.8517 | 1.835 | 0.2666 |
| cg22515201 | –0.791 | 6.07E-04 | – | – | –7.309 | 0.3455 | 2.370 | 0.4033 |
| cg24823993 | –0.355 | 2.29E-03 | – | – | –3.080 | 0.6645 | 0.682 | 0.7930 |

**Supplementary Table S4: Association between CpG methylation of top sites and RNA expression of associated genes**

| CpG Site | Chr | Gene | Beta* | P* | $R^2$ |
|---|---|---|---|---|---|
| cg11875268 | 3 | *WWTR1* | −23.202 | 0.375 | 0.043 |
| cg16200496 | 12 | *SMUG1* | 1.509 | 0.862 | 0.015 |
| cg22515201 | 19 | *NFIX* | −79.627 | 0.267 | 0.015 |
| cg24823993 | 22 | *PLA2G6* | 1.754 | 0.686 | 0.053 |
| cg25771041 | 22 | *NHP2L1* | −146.200 | 0.006 | 0.089 |

*N* = 410 for all analyses.
*Adjustment covariates: Sex, race, age at diagnosis, cell type, *KRAS* mutation, *EGFR* mutation status.

**Supplementary Figure S1: Cell type-specific dose-response relationships for externally validated CpG sites by M-values and smoking pack years.** M-values are logit transformed effect estimates. As the M-value approaches negative infinity, the estimate approaches zero. As the M-value approaches positive infinity, the estimate approaches 1. The solid black line represents the model of the effect estimate (M-value) by Pack years (smoking). The red, dotted line represents the upper and lower 95% confidence bounds. Figures are presented as Adenocarcinoma (ACS) and squamous cell carcinoma (SQC) sequentially per CpG site.

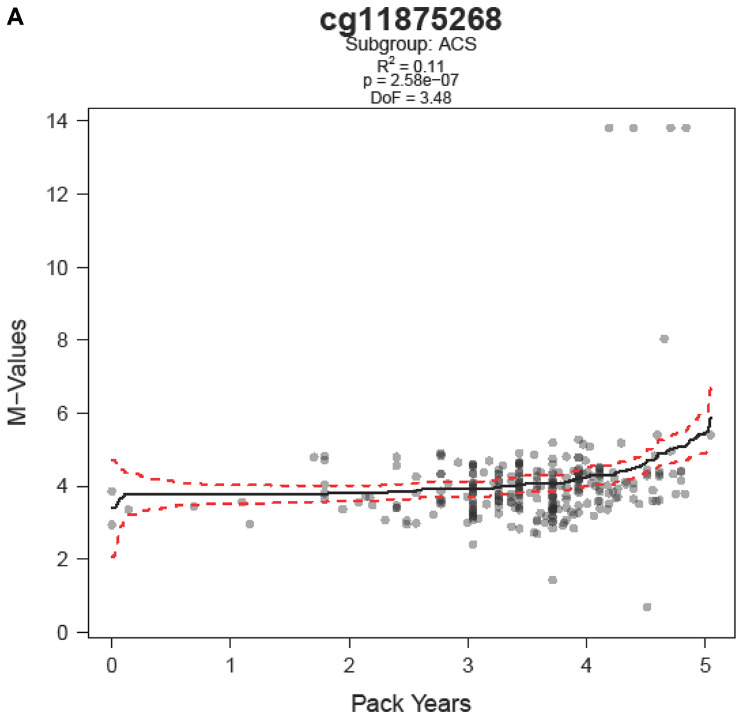


**Supplementary Figure S1A: Smoking and methylation dose-response at cg11875268 in *SMUG1* for Adenocarcinoma-only cases.** There appears to be a significant, positive dose-response relationship between pack-years and M-values. Higher smoking pack-years are associated with increased M-values ($p$ = 2.58E-7). The trend line suggests higher smoking exposure levels impose positive effects on M-values. The multivariate linear regression model restricted to adenocarcinoma cases adjusting for smoking status and all confounding covariates explains only some of the total variability in methylation at this site ($R^2$ = 0.11).

B

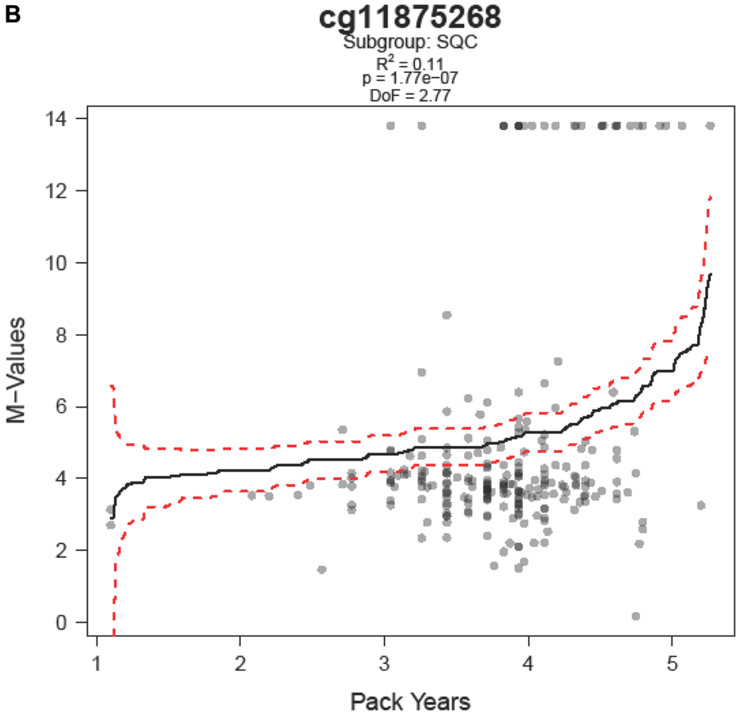


**Supplementary Figure S1B: Smoking and methylation dose-response at cg11875268 in *SMUG1* for Squamous cell carcinoma-only cases.** There appears to be a significant, positive dose-response relationship between pack-years and M-values. Higher smoking pack-years are associated with increased M-values ($p$ = 1.77E-7). The trend line suggests higher smoking exposure levels impose positive effects on M-values. The multivariate linear regression model restricted to squamous cell cases adjusting for smoking status and all confounding covariates explains only some of the total variability in methylation at this site ($R^2$ = 0.11).

C

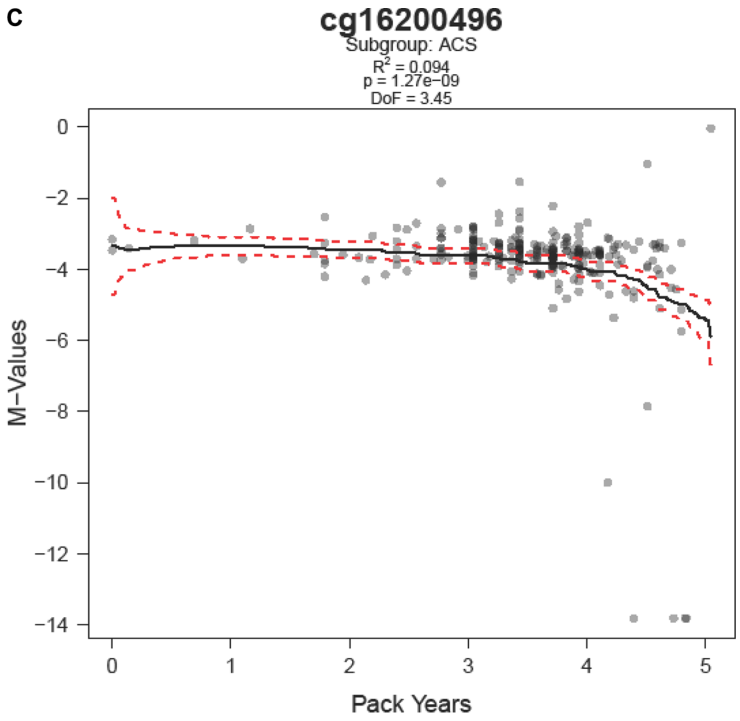


**Supplementary Figure S1C: Smoking and methylation dose-response at cg16200496 in *NFIX* for Adenocarcinoma-only cases.** There appears to be a significant, negative dose-response relationship between pack-years and M-values. Higher smoking pack-years are associated with decreased M-values ($p$ = 1.27E-9). The trend line suggests higher smoking exposure levels impose negative effects on M-values. The multivariate linear regression model restricted to adenocarcinoma cases adjusting for smoking status and all confounding covariates explains only some of the total variability in methylation at this site ($R^2$ = 0.094).

D

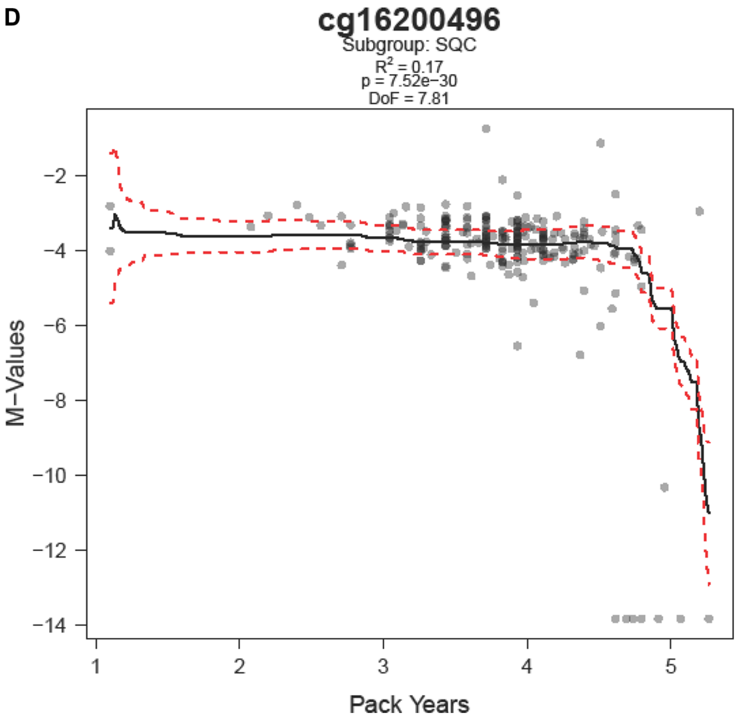


**Supplementary Figure S1D: Smoking and methylation dose-response at cg16200496 in *NFIX* for Squamous cell carcinoma-only cases.** There appears to be a highly significant, negative dose-response relationship between pack-years and M-values. Higher smoking pack-years are associated with strongly decreased M-values ($p$ = 7.52E-30). The trend line suggests higher smoking exposure levels impose negative effects on M-values. The multivariate linear regression model restricted to squamous cell cases adjusting for smoking status and all confounding covariates explains only some of the total variability in methylation at this site ($R^2 = 0.17$).

E

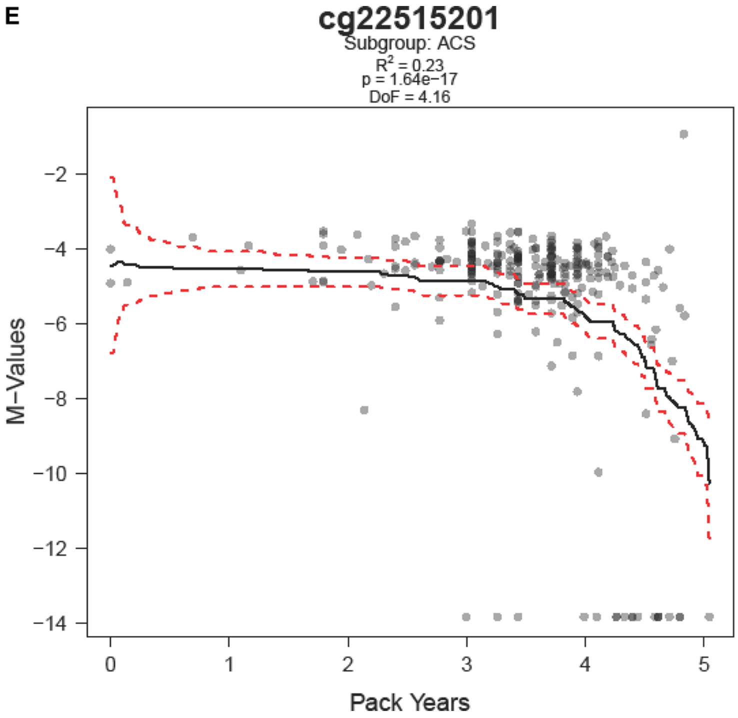


**Supplementary Figure S1E: Smoking and methylation dose-response at cg22515201 in *PLA2G6* for Adenocarcinoma-only cases.** There appears to be a highly significant, negative dose-response relationship between pack-years and M-values. Higher smoking pack-years are associated with strongly decreased M-values ($p$ = 1.64E-17). The trend line suggests higher smoking exposure levels impose negative effects on M-values. The multivariate linear regression model restricted to adenocarcinoma cases adjusting for smoking status and all confounding covariates explains only some of the total variability in methylation at this site ($R^2$ = 0.23).

F

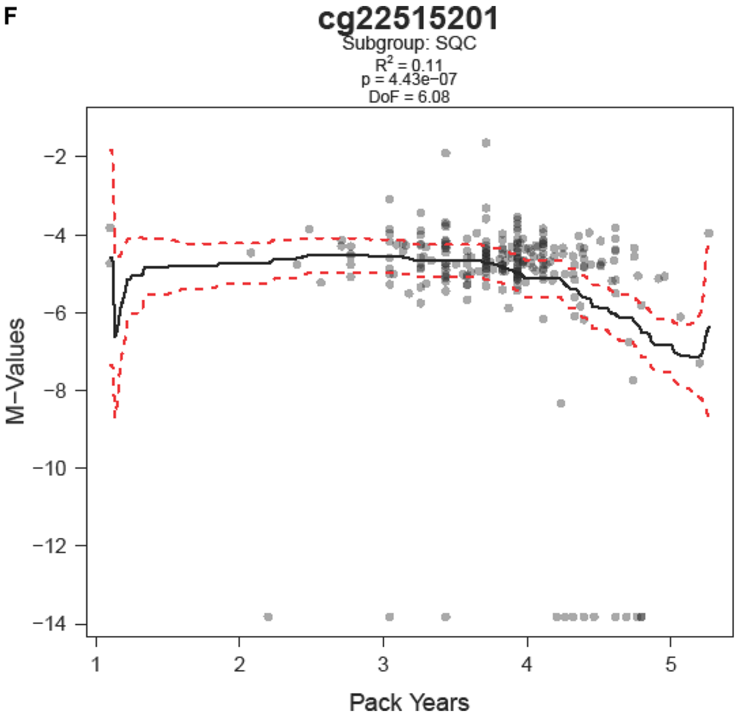


**Supplementary Figure S1F: Smoking and methylation dose-response at cg22515201 in *PLA2G6* for Squamous cell carcinoma-only cases.** There appears to be a significant, negative dose-response relationship between pack-years and M-values. Higher smoking pack-years are associated with decreased M-values ($p = 4.43E\text{-}07$). The trend line suggests higher smoking exposure levels impose negative effects on M-values. The multivariate linear regression model restricted to squamous cell cases adjusting for smoking status and all confounding covariates explains only some of the total variability in methylation at this site ($R^2 = 0.11$).

G

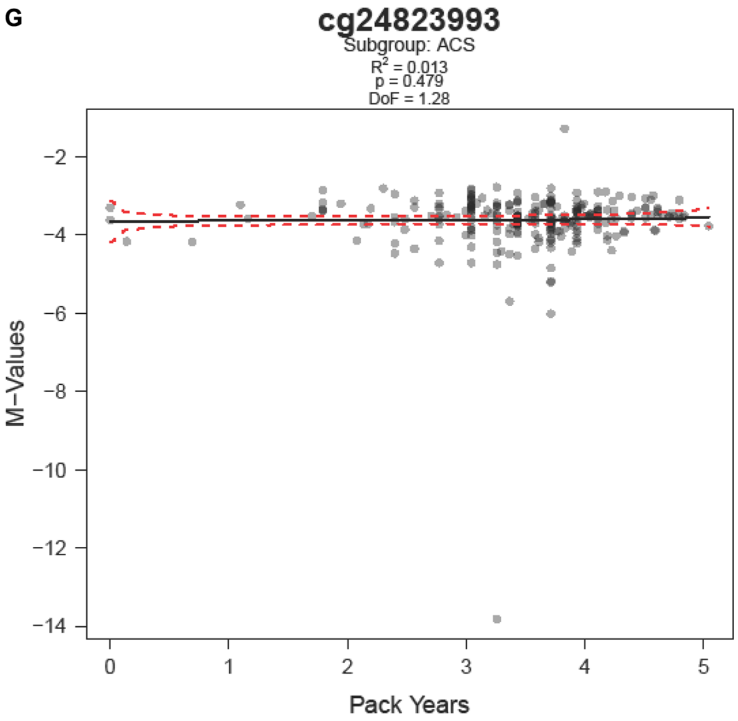


**Supplementary Figure S1G: Smoking and methylation dose-response at cg24823993 in *NHP2L1* for Adenocarcinoma-only cases.** There appears to be a nonsignificant, dose-response relationship between pack-years and M-values. Higher smoking pack-years are not associated with M-values ($p$ = 0.479). The trend line suggests higher smoking exposure levels do not impose effects on M-values. The multivariate linear regression model restricted to adenocarcinoma cases adjusting for smoking status and all confounding covariates explains little of the total variability in methylation at this site ($R^2 = 0.013$).

H

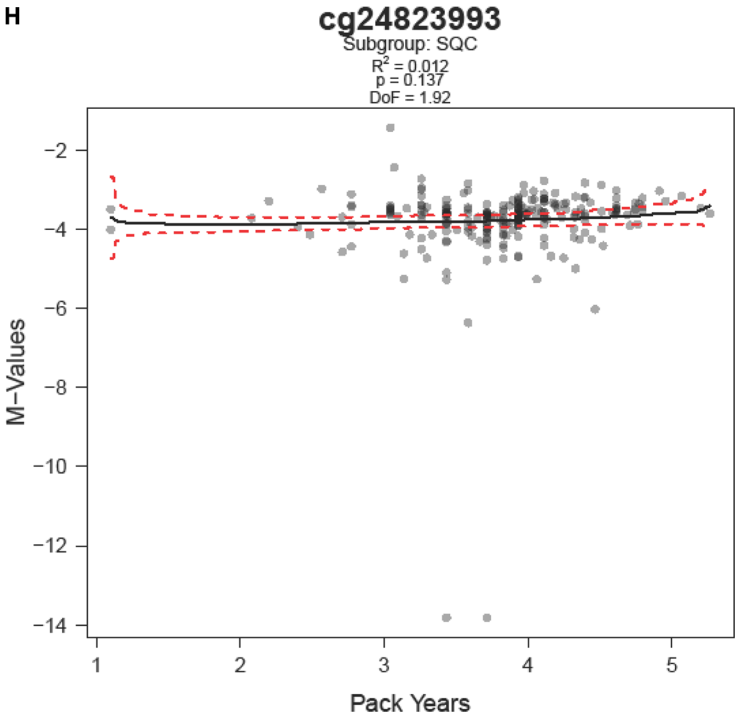


**Supplementary Figure S1H: Smoking and methylation dose-response at cg24823993 in *NHP2L1* for Squamous cell carcinoma-only cases.** There appears to be a nonsignificant, dose-response relationship between pack-years and M-values. Higher smoking pack-years are not associated with M-values ($p$ = 0.137). The trend line suggests higher smoking exposure levels do not impose effects on M-values. The multivariate linear regression model restricted to squamous cell cases adjusting for smoking status and all confounding covariates explains little of the total variability in methylation at this site ($R^2$ = 0.012).

I

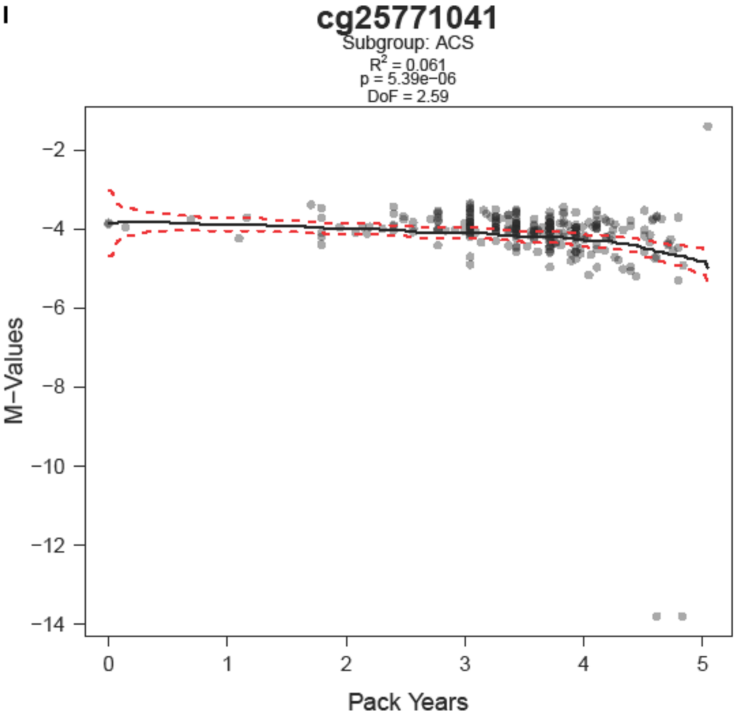


**Supplementary Figure S1I: Smoking and methylation dose-response at cg25771041 in *WWTR1* for Adenocarcinoma-only cases.** There appears to be a significant, negative dose-response relationship between pack-years and M-values, where higher values for smoking in pack years are associated with lower M-values ($p$ = 5.39E-6). The trend line also suggests higher levels of smoking exposure exert more negative effects on M-values. The multivariate linear regression in adenocarcinoma cases adjusting for smoking status cases and all confounding covariates explains only a small proportion of the total variability in the methylation patterns at this site ($R^2$ = 0.061).

J

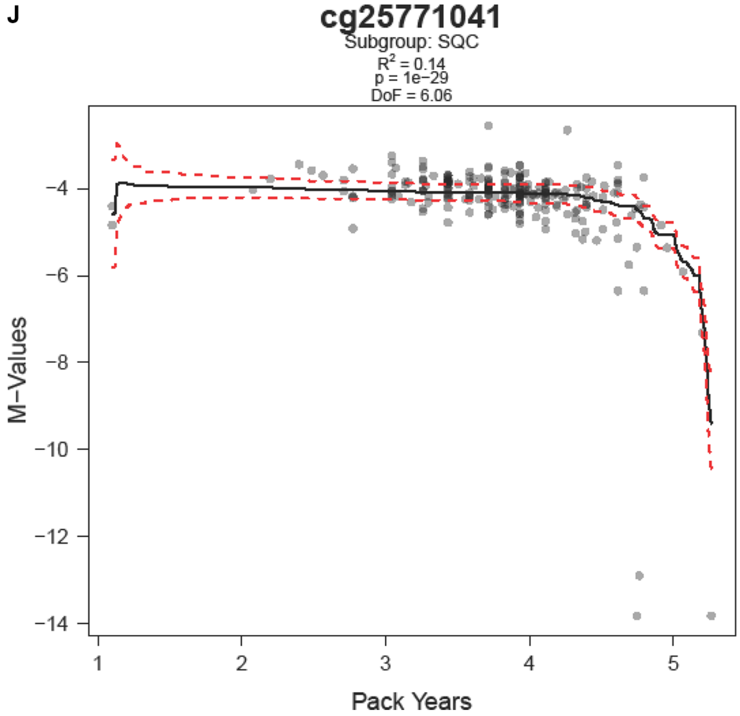


**Supplementary Figure S1J: Smoking and methylation dose-response at cg25771041 in *WWTR1* for Squamous cell carcinoma-only cases.** There appears to be a strongly significant, negative dose-response relationship between pack-years and M-values, where higher values for smoking in pack years are associated with lower M-values ($p$ = 4.06E-19). The trend line also suggests higher levels of smoking exposure exert more negative effects on M-values. The multivariate linear regression in squamous cell cases adjusting for smoking status and all confounding covariates explains only a small proportion of the total variability in the methylation patterns at this site ($R^2$ = 0.14).

**Supplementary Figure S2: Outlier sensitivity analyses for dose-response relationships of top externally validated CpG sites.** Thin-plate regression splines were used to fit dose-response relationships between smoking and CpG methylation with 1) the most extreme M-values removed and 2) the most extreme M-values substituted with the next most extreme M-values. As before, M-values are logit transformed effect estimates. The solid black line represents the model of the effect estimate (M-value) by Pack years (smoking). The red, dotted lines represent the upper and lower 95% confidence bounds. Figures are presented side-by-side with outliers removed and substituted.

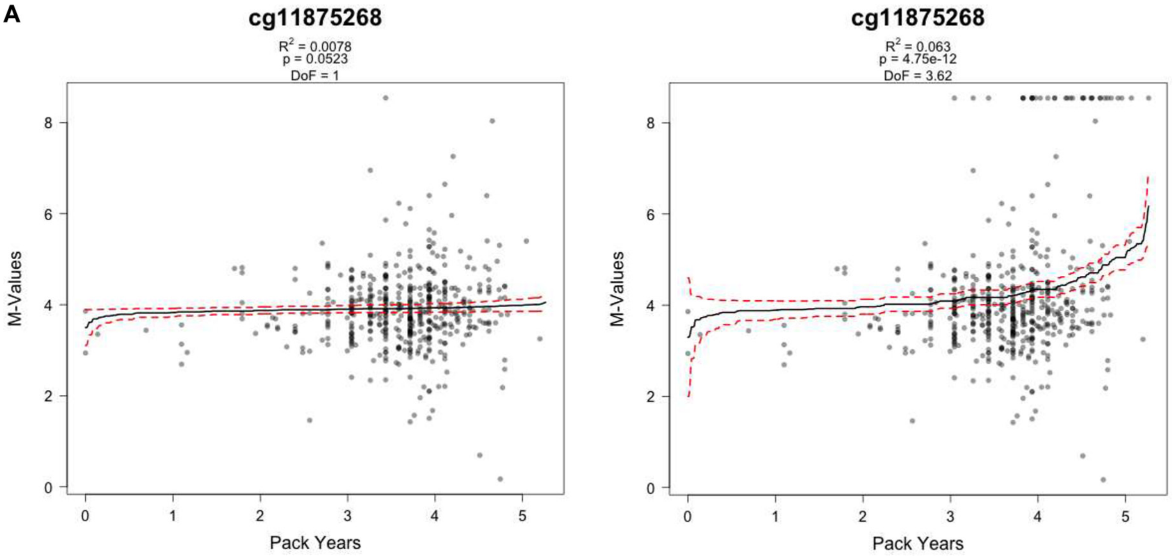


**Supplementary Figure S2A: Sensitivity of smoking and methylation dose-response at cg11875268 in *SMUG1*.** The significance of the dose-response relationship between smoking and methylation at cg11875268 is not preserved when extreme M-values are excluded from the analyses (left panel), but are preserved when they are substituted (right panel).

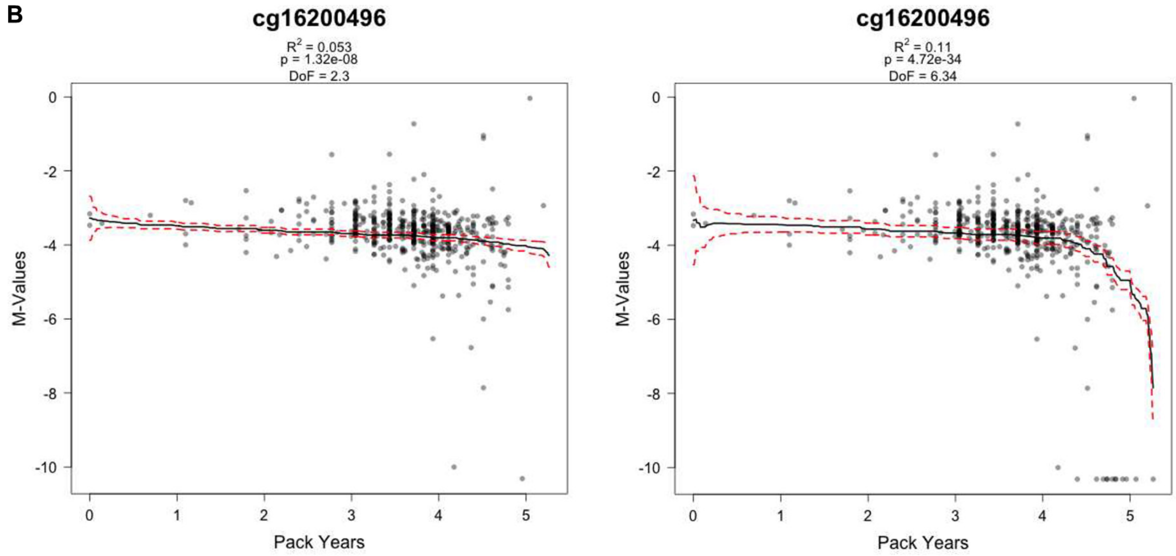


**Supplementary Figure S2B: Sensitivity of smoking and methylation dose-response at cg16200496 in *NFIX*.** The significance of the dose-response relationship between smoking and methylation at cg16200496 is preserved when extreme M-values are excluded from the analyses (left panel), as well as when they are substituted (right panel).

C

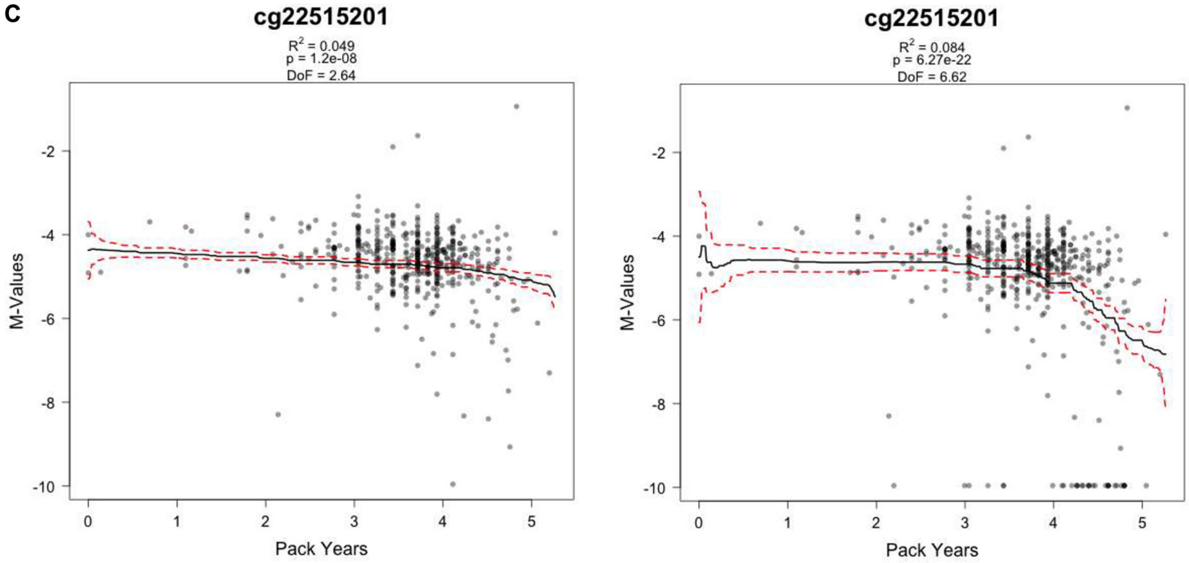


**Supplementary Figure S2C: Sensitivity of smoking and methylation dose-response at cg22515201 in *PLA2G6*.** The significance of the dose-response relationship between smoking and methylation at cg22515201 is preserved when extreme M-values are excluded from the analyses (left panel), as well as when they are substituted (right panel).

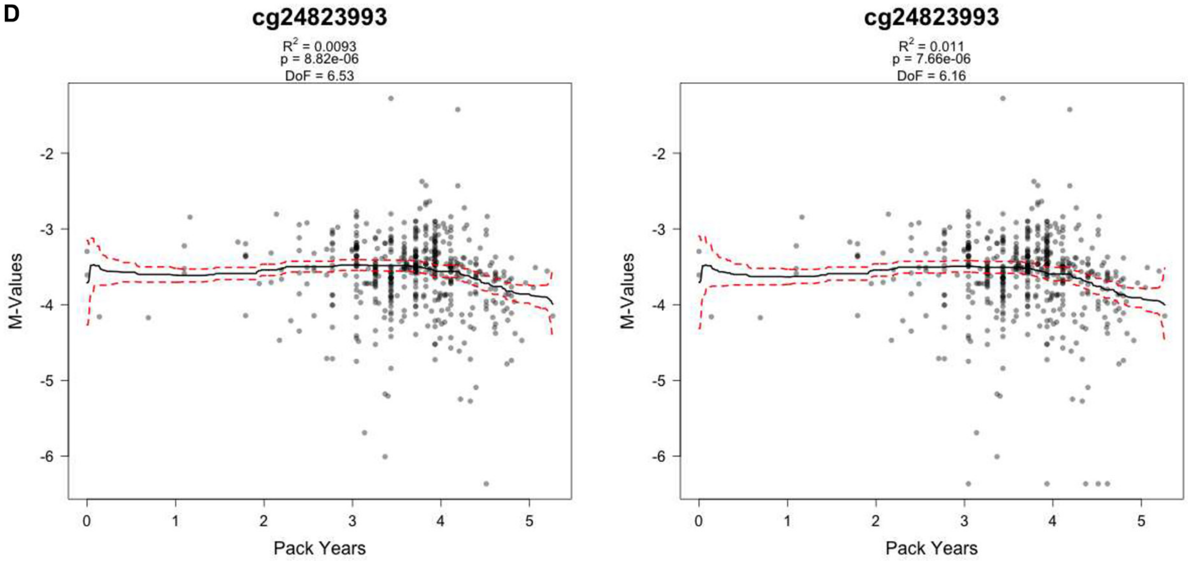


**Supplementary Figure S2D: Sensitivity of smoking and methylation dose-response at cg24823993 in *NHP2L1*.** The significance of the dose-response relationship between smoking and methylation at cg24823993 is preserved when extreme M-values are excluded from the analyses (left panel), as well as when they are substituted (right panel).

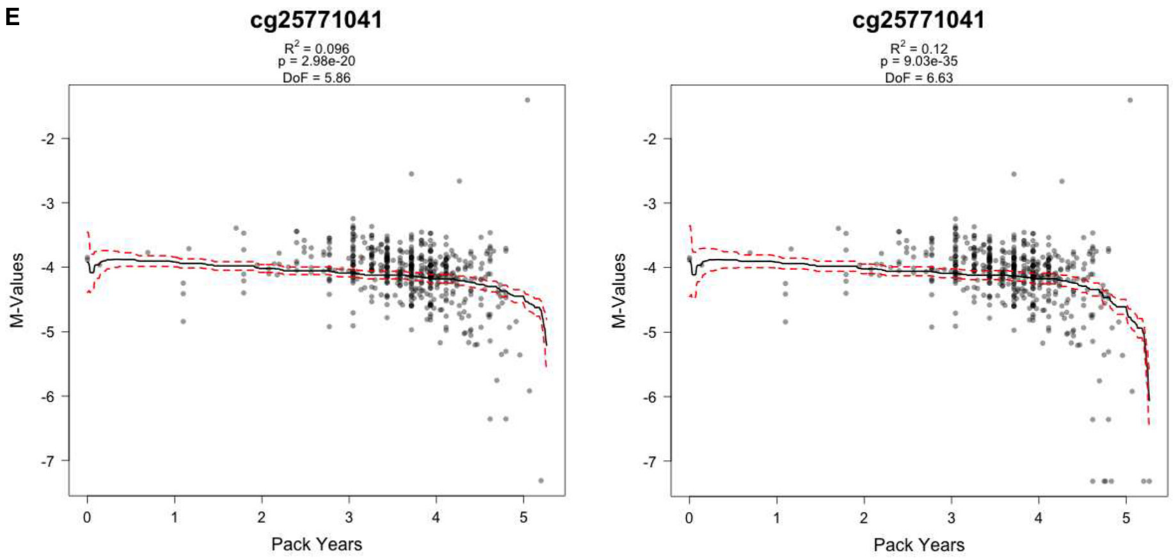


**Supplementary Figure S2E: Sensitivity of smoking and methylation dose-response at cg25771041 in *WWTR1*.** The significance of the dose-response relationship between smoking and methylation at cg25771041 is preserved when extreme M-values are excluded from the analyses (left panel), as well as when they are substituted (right panel).

**Supplementary File S1: Internally validated CpG sites.** *Beta here is the difference in methylation M-value per one-unit increase in log-transformed smoking pack-years. †Beta here is the difference in methylation M-value comparing ever smokers with never smokers. ‡Beta here is the difference in methylation M-value between current smokers and former smokers as well as between former smokers and never smokers.

**Supplementary File S2: Internally validated CpG sites-adenocarcinoma only.** *Beta here is the difference in methylation M-value per one-unit increase in log-transformed smoking pack-years. †Beta here is the difference in methylation M-value comparing ever smokers with never smokers. ‡Beta here is the difference in methylation M-value between current smokers and former smokers as well as between former smokers and never smokers.

**Supplementary File S3: Internally validated CpG sites-squamous cell carcinoma only.** *Beta here is the difference in methylation M-value per one-unit increase in log-transformed smoking pack-years. †Beta here is the difference in methylation M-value comparing ever smokers with never smokers. ‡ Beta here is the difference in methylation M-value between current smokers and former smokers as well as between former smokers and never smokers. **No adjustments were made for mutation status in the externally validated CpG sites since there was no variation in mutation status.
